# Contribution of binocular visual function and its impairment by Bangerter foils to the performance of a precision reaching, grasping and placing task in healthy adults

**DOI:** 10.3389/fnins.2025.1708514

**Published:** 2025-12-17

**Authors:** Pilar Granados-Delgado, Miriam Casares-López, José Juan Castro-Torres, Xiaoxin Chen, Ewa Niechwiej-Szwedo

**Affiliations:** 1 Department of Optics, Faculty of Sciences, University of Granada, Granada, Spain; 2 Department of Kinesiology and Health Sciences, Faculty of Health, University of Waterloo, Waterloo, ON, Canada

**Keywords:** binocular vision, visual impairment, stereoacuity, contrast sensitivity, upper limb movements, kinematics, latency, phoria

## Abstract

**Background:**

Upper limb movement execution is affected in patients with vision impairments resulting from ocular pathologies or binocular anomalies. Deficits in motor performance have been correlated with reduced contrast sensitivity, phoria, and stereoacuity.

**Aim:**

To evaluate the effects of increased straylight induced by Bangerter foils on binocular visual function and examine the corresponding impact on eye-hand kinematics during a high precision reaching, grasping and placement task (i.e., bead threading).

**Methods:**

Twenty-one healthy participants, aged 21–41 years, participated in this experimental study. Visual function assessment included visual acuity (VA), contrast sensitivity (CS), stereoacuity (SA), phoria, and fusional vergences (FV). Vision was impaired experimentally using Bangerter foils 0.4. Participants performed a bead threading task under three randomized viewing conditions: binocular baseline (BB; normal vision), binocular viewing with a foil over the dominant eye (FDE), and foils over both eyes (FBE). Eye and hand movements were recorded using an eye tracker and a motion capture camera.

**Results:**

Visual functions (VA, CS, and stereoacuity) were impaired when viewing with the Bangerter foils. Pairwise comparison between viewing conditions confirmed statistically significant differences between BB, FDE and FBE for mid to high spatial frequencies (6, 12 and 18 cycles per degree; *p* < 0.05). Break points for positive and negative fusional reserves were impaired when interocular differences increased (i.e., FDE condition). The impairment did not have any impact on phoria. Results from the kinematic analysis revealed threading duration was prolonged using Bangerter foils on both eyes (FBE condition). In contrast to the hypothesis, visual deficit due to foils did not affect any other kinematic measures. However, results from mixed model analyses revealed that individual differences in phoria and fusional vergence had the most significant influence on reaching performance. Specifically, a larger exophoria magnitude was associated with longer reach duration, lower peak velocity and prolonged total time to complete the task (*p* < 0.05).

**Conclusion:**

Inducing short-term deterioration through the alteration of contrast sensitivity at high spatial frequencies without a clinical deterioration in stereopsis does not have a significant impact on precision grasping and placing movements, in a repetitive manual task. Notably, this study demonstrated that phoria impacts the execution of reaching movements.

## 1 Introduction

A multifaceted relationship is unveiled in the context of eye-hand coordination, which is characterized by the interplay between the visual, ocular and manual motor systems. The accuracy, precision and speed of fine motor skills such as reaching, grasping and placing is intrinsically linked to an individual’s capacity to discern sensory information from the environment and to orchestrate the appropriate ocular and manual responses (Niechwiej-Szwedo et al., 2011; Niechwiej-Szwedo et al., 2023). Upper limb reach and grasp movements are performed more efficiently during binocular viewing (Niechwiej-Szwedo et al., 2020; Servos et al., 1992; Melmoth and Grant, 2006) by children and adults with normal vision (Servos et al., 1992; Gnanaseelan et al., 2014; Read et al., 2013; Granados-Delgado et al., 2024). On the other hand, individuals with impaired binocular vision due to amblyopia and strabismus demonstrate deficits on prehension tasks (Grant and Conway, 2015; Kelly et al., 2021; Niechwiej-Szwedo, 2024). Upper limb movement execution is also affected in patients with vision impairments resulting from other ocular pathologies or binocular anomalies (Niechwiej-Szwedo et al., 2020; Grant and Conway, 2015; Rakshit et al., 2024; Verghese et al., 2016). For instance, the performance of manual tasks was evaluated in patients with central visual impairment due to macular degeneration (Verghese et al., 2016; Pardhan et al., 2011). Results demonstrated prolonged reaching and grasping duration when compared to age-matched participants with normal vision. Furthermore, a greater number of kinematic indices (i.e., longer total time, deceleration duration, and time to maximum grip aperture) were correlated with reduced contrast sensitivity rather than visual acuity (Pardhan et al., 2011). Notably, another study demonstrated that a binocular advantage in peg-placement time was significantly correlated with stereoacuity (Verghese et al., 2016). Aging is associated with reduced visual function including acuity, contrast sensitivity, stereoacuity, phoria and near point of convergence, which has been associated with self-reported disability (Elliott et al., 1990; Rubin et al., 2001; Lennerstrand and Ahlström, 1989; Leat et al., 2013). While self-reported disability offers an important perspective on the patient’s perception of their function, such measures could be augmented with objective visuomotor tests. However, no objective tests are currently available in clinic. Developing such tests requires research to understand how visual impairments affect various aspects of motor control.

Several studies to date have manipulated various aspects of visual functions to evaluate their impact on motor task performance (Sheppard et al., 2021; Piano and O’Connor, 2013; González-Alvarez et al., 2007; Abraham et al., 2024). For example, prisms and convex lenses have been used to disrupt stereopsis and vergence (Melmoth and Grant, 2006; Melmoth et al., 2007; Melmoth et al., 2009). Experimental results revealed that reducing stereopsis using convex lenses impaired the execution of grasping, while disrupting fusional vergence using base out or base in prisms was associated with a misestimation of target distance and reaching errors (Niechwiej-Szwedo et al., 2020; Melmoth and Grant, 2006; Melmoth et al., 2007; Melmoth et al., 2009). Moreover, individual’s magnitude of phoria induced during monocular viewing is associated with endpoint error during open-loop pointing (i.e., when the arm and hand cannot be seen during the movement) (Ono and Weber, 1981). However, it is currently unknown if latent deviation of the visual axes (i.e., phoria) contributes to the dynamic control upper limb reaching movements in adults.

Bangerter foils can be used to induce visual impairment in visually normal adults (Sheppard et al., 2021; Chen et al., 2015; Odell et al., 2008; Zhang et al., 2022; Williamson et al., 2021; Martino et al., 2022; Martino et al., 2023). Foils are also used for amblyopia treatment to reduce vision in the fellow eye instead of patching (Agervi et al., 2009; Abrams et al., 2011; Lee and Kim, 2021). The blur from Bangerter foils is qualitatively different from defocus (Pérez et al., 2010) because it induces augmentation in straylight resulting in a deterioration in retinal image quality and, therefore, a decline in visual acuity and contrast sensitivity (Castro-Torres et al., 2021). According to previous studies, Bangerter foil cleanly attenuates the higher spatial frequencies to which the fovea is more sensitive. In contrast, the effect of defocus, using power lenses, on various spatial frequencies and distances is much more complex (Abrams et al., 2011). Using defocusing lenses disrupts the synergy between accommodation and vergence. In contrast, Bangerter foils increase the straylight, which uniformly increases central and peripheral blur, but not defocus blur. These effects mimic the experience of cataracts, comparable to the Lens Opacity Classification System III (LOCS III) grades in cataract classification (Sheppard et al., 2021; Martino et al., 2022; Martino et al., 2023). The impact of Bangerter foils on binocular visual performance is contingent on the application of the foil to either one eye or both eyes. Specifically, studies demonstrated that a mild monocular visual impairment (i.e., a Bangerter foil placed over one eye) disrupts stereopsis to a greater degree than placing the foil over both eyes (Sheppard et al., 2021). Therefore, even a mild uniocular disruption in visual acuity or contrast sensitivity may impact binocular integration and stereoacuity. These findings contrast with the effects observed for visual acuity and contrast sensitivity, where the impairment is more pronounced when the Bangerter foil is applied binocularly (Sheppard et al., 2021; Martino et al., 2023). As mentioned above, binocular vision plays a crucial role in planning and executing prehension movements. Sheppard et al. demonstrated that Bangerter foils caused decreased task performance in water pouring, peg placing, and aiming tasks, confirming that unilateral and bilateral visual impairment has functional implications (Sheppard et al., 2021). This also highlights the need to establish a deeper understanding of the relationship between visual function and visuomotor performance. Gaining such insights could support the development of objective assessments to complement existing self-report methods, offering a more comprehensive approach to evaluating the impact of visual impairments on disability (Kelly et al., 2025).

.The impact of interocular difference on visual performance has been previously examined, along with the effects on everyday activities such as driving or pouring water (Sheppard et al., 2021; Ortiz-Peregrina et al., 2022). However, these studies did not provide a detailed examination of visuomotor control using eye and limb motion tracking. Recording eye and hand kinematics could reveal insights into movement planning and execution, and a deeper understanding about the role of vision in sensorimotor control. Therefore, the present study evaluated the effect of impaired vision, introducing augmentation of straylight, on fine motor skills using a precision grasping and placement task, which relies on binocular vision (Niechwiej-Szwedo et al., 2020; Niechwiej-Szwedo et al., 2021). A relatively mild vision impairment was induced with Bangerter 0.4 foils either binocularly (foils placed in front of both eyes) or monocularly (a foil placed over one eye). The impact of Bangerter foils on binocular visual function such as phoria, fusional vergences and stereopsis was analyzed. Based on previous findings, it was hypothesized that binocular impairment would be associated with reduced visual acuity and contrast sensitivity. On the other hand, it was expected that stereoacuity thresholds would be higher under monocular deterioration condition due to the interocular differences in visual acuity and contrast sensitivity. Moreover, the decline in visual function was expected to affect visuomotor performance, resulting in slower movement execution. Specifically, greater phoria and reduced fusional vergence ranges were expected to correlate with slower reach velocities, while poorer stereoacuity (higher thresholds) would likely extend the duration of grasping actions.

## 2 Materials and methods

### 2.1 Participants

Twenty-one participants (16 women and 5 men; age: 26.1 ± 7.1 years) were recruited from the University of Waterloo and the local community. The inclusion criteria were: age between 18 and 41 years (to avoid presbyopia), normal or corrected to normal visual acuity (VA no worse than 0.0 logMAR at both near and distance), normal binocular vision, and normal stereoacuity (no worse than 40 s of arc). The exclusion criteria included any ocular or general disease, presbyopia, or any history of diagnosed neurological conditions such as concussion.

### 2.2 Procedures

The study protocol was approved by the University of Waterloo Research Ethics Board (REB # 46326). Participants signed the consent form before starting the collection procedure.

Participants were asked to wear their habitual prescription contact lenses during testing. The session began with a complete refractive and binocular visual examination, which included retinoscopy to check the refractive status, measurements of accommodative function (amplitude of accommodation [AA], and accommodative response [AR]), and measurement of planar fusion using Worth 4 Dot lights. This assessment was used to ensure that participants met the inclusion criteria.

Afterwards, the experiment was conducted in three blocks, which were randomized between participants: a control condition (normal binocular vision [BB]), and two conditions in which vision was degraded using 0.4 Bangerter foils (Ryser Optik, St Gallen, Switzerland), corresponding to 0.4 in decimal notation (20/50 in Snellen, or 0.4 in logMAR), whose negative effect on visual quality has been demonstrated in several studies (Martino et al., 2022; Pérez et al., 2010; Castro-Torres et al., 2021). The foils were adhered directly onto plano ophthalmic lenses, which were mounted in identical optical frames. Bangerter foil 0.4 is formed by microbubbles (around 3.76 bubbles/mm^2^) which produce the image degradation (Castro-Torres et al., 2021). Bangerter foils were placed either on both lenses, thus affecting both eyes (FBE), or over one lens in front of the dominant eye (FDE).

The visual functions described below were assessed binocularly under the three experimental conditions (BB, FBE, FDE). Following the visual assessment participants completed the bead threading task described in the next subsection.

#### 2.2.1 Visual acuity

The measurement of near and distance visual acuity (VA) was conducted using the Saladin Near Point Balance Card™ (Bernell, Michigan College of Optometry, USA) and the Runge Sloan Letter Pocket Near Vision Card (Good-Lite® Co. Paul Runge, USA) at 40 cm, and the Bailey Lovie Chart #5 at 3 m (National Vision Research Institute of Australia), respectively.

#### 2.2.2 Stereoacuity

Stereopsis was evaluated using two tests: the Frisby test, which shows real depth (i.e., non-dissociative test), and the Randot stereoacuity test (Stereo Optical Company, Chicago USA), which is a polarized test (i.e., a dissociative test). The tests also differ in the range of disparity values that can be measured, with Frisby ranging between 5 to 600 s of arc, and Randot ranging from 20 to 400 s of arc. The publisher’s guidelines were followed to administer the tests.

#### 2.2.3 Phoria

Phoria was determined using the alternating cover test performed at 40 cm. The value of the prism when the eye correction movement was observed was recorded. Esophoria was indicated with a positive value and exophoria with a negative value, using prism bars base out and in, respectively.

#### 2.2.4 Horizontal fusional vergences

Horizontal fusional vergence (HFV) was measured using prism bars at 40 cm. The two types of horizontal fusional vergence that facilitate the maintenance of the alignment of the visual axes were measured: positive fusional vergence (PFV), in which both visual axes are displaced towards the nasal direction (prism base out), and negative fusional vergence (NFV), where visual axes are displaced in the temporal direction (prism base in). The break point and the recovery point were measured, and all values obtained were positive.

#### 2.2.5 Contrast sensitivity

Contrast sensitivity (CS) was measured at 40 cm using Gabor patches of sinusoidal gratings. CS was assessed at six spatial frequencies: 0.75, 1.5, 3, 6, 12 and 18 cycles per degree (cpd). These gratings were presented on a laptop monitor (1366×796, 15.6-inch). Participants were instructed to indicate whether the grating appeared to incline to the right, left or vertical (three-alternative forced-choice). The contrast of these gratings was systematically decreased until it could no longer be determined by the participants (from 0.93 to 0). The contrast sensitivity curve is a graphical representation of the relationship between the threshold visibility (inverse of the threshold contrast) and the spatial frequency of the stimulus. Threshold visibility is defined as the inverse of the minimum contrast value that can be perceived by an individual subject for a specific spatial frequency. The range of possible contrast sensitivities to be obtained was from 1 to 250.

#### 2.2.6 Prehension task

**Figure 1:**
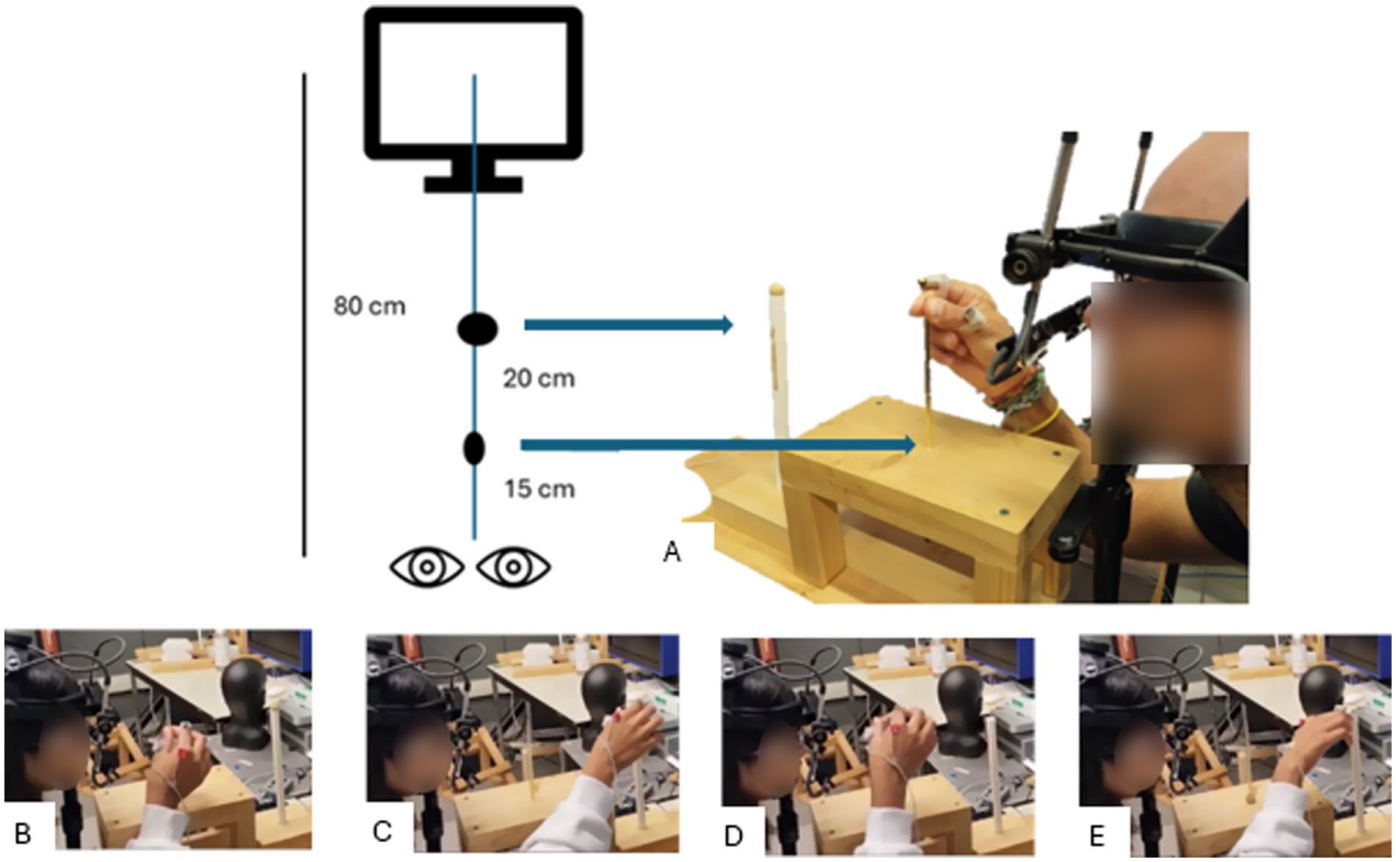
**(A)** Experimental setup for the bead threading task. Task sequence: **(B)** At the start of each trial the participant pinched the needle between their fingers; **(C)** Upon hearing an auditory signal, the participant reached towards the bead and grasped it; **(D)** Next, the participant transported the bead back to the needle and threaded the bead onto it; **(E)** Finally, the participant pointed to the bead holder, indicating completion of the task.

Visuomotor control was assessed using a bead threading task. The experimental setup is illustrated in Figure 1A. A vertical needle (height: 12 cm; diameter: 0.3 cm) was placed 15 cm in front of the chinrest, and a bead holder was placed 20 cm in front of the needle, as in previous studies (Niechwiej-Szwedo et al., 2020). This set up ensured a comfortable reaching distance for all participants. A bead (diameter: 1.0 cm; hole diameter: 0.5 cm) was placed on the holder by the experimenter. All participants initiated the task by pinching the needle between their index finger and thumb. The commencement of the bead threading task was indicated by an auditory signal, which served as a “Go” signal. The task involved the following actions: reaching towards the bead, grasping it, transporting it towards the needle, and placing it on the needle (Figures 1B–F, respectively). Each participant completed 45 trials total with 15 trials for each viewing condition (control [BB], foil over one eye [FDE], foils over both eyes [FBE]), performed in randomized blocks.

##### 2.2.6.1 Hand kinematics recording

Hand kinematics were recorded at 250 Hz using a motion capture camera (OptoTrak 3D Investigator, NDI, Waterloo, ON, Canada) while participants performed the motor task involving reaching, grasping and threading a bead onto a needle (Niechwiej-Szwedo et al., 2020).

Two infrared markers were placed at the distal phalanx of the thumb and index finger of the preferred hand. The OptoTrak system was calibrated such that positive x (azimuth) was to the right, positive z (depth) was towards the screen, and positive y corresponded with elevation.

#### 2.2.7 Eye movement recording

Eye movements were recorded at 250 Hz with EyeLink II (SR Research Ltd., Ottawa, ON, Canada), a video-based eye tracker using pupil–corneal reflection tracking. The system was calibrated using a nine-point grid before each block (i.e., before each viewing condition). Validation was performed and accepted when the tracking error was less than 1 degree. The eye tracking recording was synchronized with the OptoTrak motion capture system using a TTL pulse (Zafar et al., 2023).

### 2.3 Data analysis

The raw kinematic data were filtered using a dual pass Butterworth filter with a low pass cut-off frequency of 15 Hz for the hand data and 80 Hz for the eye data. The velocity trajectory was obtained using two-point differentiation. Next, a custom MATLAB script was used to plot the position and velocity trajectory, and all trials were visually inspected to ensure accurate detection of movement initiation and termination. Trials with missing marker data were removed from analysis (36.6% of trials). The total number of trials per visual condition was 315, and the number of useful trials was found to be 199, 212 and 187 for the BB, FBE and FDE conditions, respectively. The high rate of trial discard was attributed to the necessity of ensuring that signals from EyeLink and OptoTrak were both viable during a single trial.

Movement onset was detected when finger velocity exceeded 30 mm/s for 20 ms, and movement termination was detected when velocity dropped to 100 mm/s. The following variables were defined based on the hand velocity trajectory: *reach to bead duration* (defined as the interval from reach initiation to reach termination); *grasp duration* (defined as the interval from reach termination to when the subsequent reach towards the needle was initiated); *reach to needle duration* (defined as the interval following grasping when the reach towards the needle was initiated to the end of that reach); *thread duration* (interval following reach termination to when the hand moved away from the needle after the bead was placed on it); *reach to bead* and *reach to needle peak velocity* (defined as the maximum velocity along the depth direction when reaching to grasp the bead and towards the needle, respectively), and *total time* to complete the bead task, which was calculated as the time from reach onset to when the threading was completed.

The eye movement analyses focused on the first movement towards the bead. The eyes moved towards the bead to guide the grasping action. Temporal eye-hand coordination was assessed by calculating the latency difference between eye and hand responses when reaching to the bead. A positive value means the eye movement was initiated before the hand movement. Conversely, a negative value means the hand movement was initiated before the eyes.

Statistical analyses were performed using SPSS 28.0 (SPSS Inc., Chicago, IL, USA). Means and standard deviations (or medians and interquartile ranges in case of skewed data) were used for descriptive analyses. Normality was assessed by the Shapiro–Wilk test. In order to analyze the effect of Bangerter foils on vision, a Friedman test and a one-way analysis of variance (ANOVA) were performed, respectively, in the case of non-normal and normal distributions. Pairwise comparison with Bonferroni correction was also performed to determine the difference between the three viewing conditions (BB, FBE, FDE). The requirement for sphericity was assessed using the Mauchly’s test of sphericity which was *p* > 0.05 for all analyses.

To analyze the association between kinematic metrics and visual functions, a linear mixed model (LMM) and a generalized mixed model (GLMM) with repeated measures were performed in the case of normal and non-normal variable distributions, respectively. The models were constructed with hand and eye kinematic variables as the dependent variables (reach-to-bead, grasp, reach-to-needle and thread durations, reach peak velocity, and eye-hand latency difference). Viewing conditions (FDE and FBE) were included as the repeated factor (fixed effect). Participant characteristics (age and visual function parameters: CS mean, VA near, phoria near, stereoacuity measured using Frisby test, PFV and NFV breakpoints) were included as predictors. Participant was modelled as a random factor.

## 3 Results

### 3.1 Visual assessment

The mean spherical optical correction was −3.39 ± 2.22 D and cylinder −0.94 ± 0.37 D. The mean accommodative amplitude (AA) was 11.47 ± 2.50 D, and the mean accommodative response using monocular estimation method (MEM) was 0.45 ± 0.24 D, a value within the normal range (Scheiman and Wick, 2019). All participants had an AA within the normal range for their age (Scheiman and Wick, 2019) and had planar fusion (detected 4 images using Worth 4 Dot lights).

**Figure 2:**
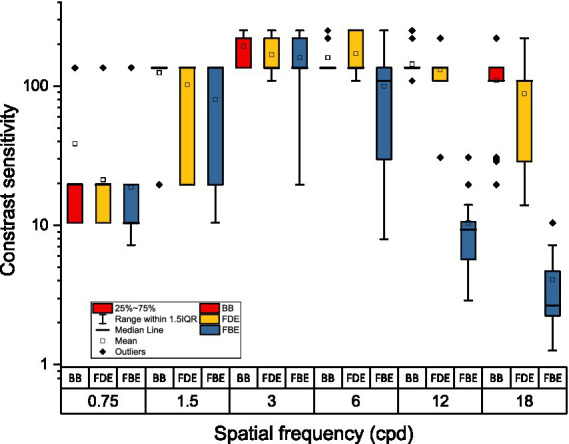
Boxplots showing binocular contrast sensitivity at six spatial frequencies measured for the three viewing conditions: binocular control (BB), Bangerter foils in the dominant eye (FDE), and Bangerter foils in both eyes (FBE).

The results of the descriptive analysis for each visual function under the three viewing conditions (BB, FBE and FDE) are presented in Table 1 and Figure 2, alongside the statistical output. Results from the visual function assessment (i.e., VA, mean CS -calculated as the average across the spatial frequencies tested, stereopsis, phoria, and FV) across the experimental conditions are presented in Table 1. It is important to note that the mean and median values for all visual functions in the control condition (BB, without Bangerter foils) were within the established normal range (Scheiman and Wick, 2019). As expected, vision was impaired by Bangerter foils. Specifically, there was a significant deterioration in VA (near and far distance), mean CS, stereoacuity (Frisby and Randot), break point of PFV, and NFV (break and recovery point). In the case of VA, although medians were equal, the Friedman test showed significant differences since this statistical analysis is not based on the medians, but on ranks within each subject. In the case of FV when the pairwise comparison was carried out, significant effect was only found for PFV and NFV break point. Furthermore, the variability between participants was found to be higher under FBE conditions. There were significant differences between BB and FBE for near and distance VA, stereoacuity (Frisby and Randot), and mean CS. The visual functions that were significantly deteriorated with FDE were distance VA, stereoacuity, break PFV and mean CS. Finally, statistical differences were observed between the two impaired viewing conditions (FDE and FBE) for VA (distance and near), NFV break, and mean CS. Phoria was not significantly different across viewing conditions.

**Table 1:** Descriptive values of mean (± SD) or median (IQR = Q_3_ –Q_1_) for the visual parameters studied in three binocular viewing conditions: baseline (BB), wearing Bangerter foils only in the dominant eye (FDE) and in both eyes (FBE).

Visual parameters		BB	FDE	FBE	Statistic (F /X^2^_(2)_)	*p*-value
Binocular Visual Acuity (VA, logMAR)	Near	−0.10 [(−0.10) –(−0.10)]	−0.10 [(−0.10) –0.00]	0.10 (0.08–0.17)	X^2^_(2)_ = 33.457 *p* < 0.001^**^	**BB-FBE: <0.001** ^**^ **FBE-FDE: <0.001** ^**^
	Distance	−0.10 [(−0.17) –(−0.09)]	−0.10 [(−0.12) –(−0.03)]	0.24 (0.17–0.31)	X^2^_(2)_ = 38.988 *p* < 0.001^**^	**BB-FDE: 0.041** ^*^ **BB-FBE: <0.001** ^**^ **FBE-FDE: 0.001** ^**^
Phoria Near (∆)		−3.0 [(−8.0) –(−1.5)]	−4.0 [(−3.5) –(−2.0)]	−3.0 [(−10.5) –(−1.5)]	X^2^_(2)_ = 1.387 *p* = 0.500	
Stereoacuity (arcsec)	Frisby	5.0 (5.0–15.0)	40.0 (30.0–75.0)	30.0 (25.0–40.0)	X^2^_(2)_ = 31.580 *p* < 0.001^**^	**BB-FDE: <0.001** ^**^ **BB-FBE: 0.003** ^*^
	Randot	20.00 (20.00–30.00)	30.00 (23.75–50.00)	35.00 (20.00–50.00)	X^2^_(2)_ = 16.941 *p* < 0.001**	BB-FDE: 0.008* BB-FBE: 0.008*
FV near (∆)	PFV Break	18.0 (16.0–35.0)	16.0 (11.0–25.0)	20.0 (14.0–37.5)	X^2^_(2)_ = 10.091 *p* = 0.006^*^	**BB-FDE: 0.033** ^*^
	PFV Recovery	14.0 (12.0–18.0)	12.0 (9.0–19.0)	18.0 (12.0–25.0)	X^2^_(2)_ = 5.912 *p* = 0.052	
	NFV Break	16.0 (14.0–18.0)	16.0 (11.0–16.0)	18.0 (15.0–21.5)	X^2^_(2)_ = 12.473 *p* = 0.002^**^	**FBE-FDE: 0.013** ^*^
	NFV Recovery	12.0 (10.0–14.0)	10.0 (8.0–14.0)	14.0 (10.0–16.0)	X^2^_(2)_ = 7.969 *p* = 0.019^*^	
Mean binocular contrast sensitivity	Near	130.30 (± 19.41)	111.50 (± 22.12)	63.61 (± 27.38)	*F*_(2,20)_ = 70.046 *p* < 0.001**	BB-FDE: 0.043* BB-FBE: <0.001** FBE-FDE: <0.001**

The comparison statistics between the experimental conditions are also included. Only significant differences are highlighted in the table (*p < 0.05; **p < 0.001). FV, Fusional vergences; PFV, Positive fusional vergences; NFV, Negative fusional vergences. BB, Binocular baseline; FDE, Foils dominant eye; and, FBE, Foils both eyes.

Table 1 shows the mean CS averaged across the spatial frequencies. A higher value indicates better contrast sensitivity. A detailed analysis was performed to assess the effect of viewing conditions separately at each of the six spatial frequencies. Figure 2 shows CS at each spatial frequency across viewing conditions. As shown in Figure 2, CS impairment is evident in the FBE condition only for the three higher spatial frequencies (i.e., 6, 12, and 18 cpd). A statistically significant difference was confirmed by a paired comparison of these three spatial frequencies between BB and FBE (*p* < 0.005; *p* < 0.001 and *p* < 0.001, respectively). There were also significant differences between FDE and FBE viewing conditions at these same three spatial frequencies (*p* < 0.001).

These findings indicate that the 0.4 Bangerter foils had a greater negative impact on CS at mid to high spatial frequencies when both eyes were impaired (FBE) compared to one-eye impairment (FDE).

### 3.2 Visuomotor performance

Kinematic results for hand and eye movements under three viewing conditions (BB, FDE and FBE) are presented in Table 2. Means and standard deviations are shown, except grasp duration, thread duration and eye-hand latency difference, for which medians and interquartile ranges are shown instead because these data were not normally distributed.

**Table 2:** Mean (±SD) and median (IQR = Q3 –Q1) values for the bead threading task in the three experimental conditions: baseline (BB), with Bangerter foil on both eyes (FBE) and on the dominant eye (FDE).

Hand movement	Viewing condition			Statistic *p*-values
	BB	FDE	FBE	(F /X^2^_(2)_)
Reach-to-Bead Duration (ms)	492.79 (± 48.54)	482.17 (± 53.30)	486.55 (± 58.24)	*F*_(2)_ = 1.281 *p* = 0.289
Reach-to-Bead Peak Velocity (mm/s)	702.44 (± 93.21)	732.25 (± 92.46)	713.80 (± 93.71)	F _(2)_ = 4.141 *p* = 0.023*
Grasp Duration (ms)	200.00 (138.45–251.75)	202.46 (135.08–241.60)	193.33 (148.77–252.30)	X^2^_(2)_ = 0.418 *p* = 0.661
Reach-to-Needle Duration (ms)	469.02 (± 53.17)	463.51 (± 44.92)	461.84 (± 51.97)	F _(2)_ = 0.489 *p* = 0.617
Reach-to-Needle Peak Velocity (mm/s)	790.68 (± 146.10)	796.45 (± 120.89)	775.12 (± 133.75)	F _(2)_ = 0.871 *p* = 0.426
Thread Duration (ms)	341.71 (305.12–439.60)	365.54 (331.59–433.15)	423.69 (338.67–520.15)	X^2^_(2)_ = 3.787 *p* = 0.031*
Total Time (ms)	1538.30 (± 183.72)	1540.24 (± 200.02)	1594.77 (± 213.01)	F _(2)_ = 1.611 *p* = 0.214
Eye-hand Latency Difference (ms)	131.50 (107.75–202.10)	124.44 (89.34–225.40)	127.67 (78.31–207.00)	X^2^_(1.605)_ = 0.191 *p* = 0.779

The comparison statistics between the experimental conditions are also included. *p < 0.05. BB, Binocular baseline; FDE, Foils dominant eye; and, FBE, Foils both eyes.

Reach peak velocity is reported in absolute values where higher peak velocity signals more efficient reaching. Positive value for eye-hand latency difference means that eye started before the hand. Significant impairment using Bangerter foils is also shown in Table 2.

The total time to perform the bead threading task was shorter during the binocular control condition (BB) in comparison to the foil in both eyes (FBE) condition, which was due to shorter thread duration (X^2^_(2)_ = 3.787; *p* = 0.031). There was also a significant effect for reach-to-bead peak velocity (*F*_(2, 19)_ = 4.141; *p* = 0.023). *Post hoc* tests revealed a difference between BB and FDE conditions, where peak velocity was lower in BB than in the FDE condition.

### 3.3 Association between binocular visual function and bead threading performance

Given the numerous visual and kinematic variables involved, the association between kinematic metrics and visual functions was investigated using a linear mixed model. The model included 378 data points: 21 subjects x 3 viewing conditions x 6 visual functions as covariates.

Hand and eye kinematic measures (reach-to-bead, grasp, reach-to-needle, and thread durations, reach peak velocity, and eye-hand latency difference) were used as the dependent variable, with viewing conditions (FDE and FBE) as the repeated (fixed) factor. Participant characteristics including age and visual function parameters, such as mean CS, near VA, near phoria, Frisby-test stereoacuity, and breakpoints for PFV and NFV, were also included in the model as random effects. To facilitate the interpretation of the results, only the covariates and factors that had a significant influence on the dependent variable are summarized in Table 3. The complete models generated for each kinematic variable are included as Supplementary material.

**Table 3:** Summarized results of the linear mixed models (LMMs) and the generalized linear mixed models (GLMMs) estimates of reach, grasp, and thread durations, reach peak velocity, total time taken to complete the bead task and eye-hand latency difference.

Parameter	Coefficient	SE	df	*t*-statistic	*p*-value	[95% CI]
Reach-to-bead duration						
FDE	−29.789	11.269	28.872	−2.644	0.013*	[(−52.840) –(−6.738)]
Phoria Near	−4.450	1.226	28.103	−3.516	0.002*	[(−7.042) –(−1.858)]
Intercept	503.003	33.447	44.489	15.039	< 0.001**	[435.616–570.389]
Akaike information criterion		593.016	Bayesian information criterion			606.939

Reach-to-Bead Peak Velocity						
FDE	37.687	17.462	27.244	2.158	0.040*	[1.873–73.502]
Phoria Near	7.460	2.065	30.698	3.612	0.001*	[3.247–11.674]
PFV Break point	1.892	0.766	46.648	2.470	0.017*	[0.351–3.432]
Intercept	705.826	53.065	46.678	13.301	< 0.001**	[599.054–812.599]
Akaike information criterion		643.853	Bayesian information criterion			657.776
Reach-to-Needle Duration						
FDE	−27.679	10.007	27.016	−2.766	0.010*	[(−48.212) –(−7.146)]
FBE	−51.271	17.655	43.911	−2.904	0.006*	[(−86.853) –(−15.689)]
CS Mean	−0.475	0.164	44.955	−2.899	0.006*	[(−0.805) –(−0.145)]
Phoria Near	−5.185	0.991	25.136	−5.231	< 0.001**	[(−7.226) –(−3.144)]
NFV Break point	−2.125	0.977	44.375	−2.175	0.035*	[(−4.093) –0.156]
Intercept	546.633	31.185	49.042	17.528	< 0.001**	[483.965–609.301]
Akaike information criterion		581.203	Bayesian information criterion			595.126
Reach-to-Needle Peak Velocity						
Phoria Near	13.062	2.930	22.421	4.458	< 0.001**	[6.992–19.131]
Intercept	716.387	82.945	30.441	8.637	< 0.001**	[547.095–885.680]
Akaike information criterion		648.451	Bayesian information criterion			698.374

Total Time						
Phoria Near	−14.366	5.238	29.251	−2.743	0.010*	[(−25.075) –(−3.657)]
Intercept	1710.443	155.607	46.775	10.992	< 0.001**	[1397.362–2023.523]
Akaike information criterion		757.363	Bayesian information criterion			771.286

Only significant results are presented. The coefficients, the standard error, the *t* statistic, the *p* value and the confidence interval (CI) are reported for the variables and conditions that were significant. For the comparisons between the different categories of each factor included in the model, the binocular baseline (BB) viewing condition was selected as a reference. *p < 0.05; **p < 0.001.

At first, in the case of reach-to-bead duration, the LMM revealed that near phoria had a significant influence on reach duration. The greater the exophoria, the greater the reach duration. The second model showed the significance of phoria and positive fusional vergences (PFV break point) in the case of reach-to-bead peak velocity. Specifically, the less the exophoria and the higher PFV, the higher the reach-to-bead peak velocity.

The LMM showed that exophoria, CS and NFV break point had a significant influence on reach-to-needle duration. Lower CS values, higher exophoria and worse negative fusional vergence resulted in a longer reach-to-needle duration. The LMM showed similar results for reach-to-needle peak velocity. The smaller the exophoria magnitude, the higher the reach-to-needle peak velocity.

In the case of eye-hand latency difference, grasp and thread durations, none of the variables were found to be significant with the GLMM analysis. The results of the LMM for the total time to complete the task demonstrated a significant influence due to phoria. Specifically, a larger exophoria was associated with a prolonged execution of the bead threading task.

There were statistically significant differences with the 0.4 foils compared to BB for reach-to-bead duration, particularly for the FDE condition. The reach-to-needle duration was also affected by the foils in both conditions, FDE and FBE conditions, in which reach durations were shorter with simulated visual impairment. A statistically significant effect for reach-to-bead peak velocity was also observed for the FDE condition, where a higher value was observed.

In summary, the results revealed that phoria influenced several kinematics variables. Moreover, the analysis did not support the hypothesis that the deterioration in VA and CS caused by the foils was the most important factor determining the detrimental effect on kinematic performance in the bead threading task.

## 4 Discussion

This study assessed the effect of an experimentally induced vision impairment using Bangerter foils on binocular visual functions, and eye and hand kinematics during the performance of a precision grasping and placement task. A binocular advantage for the performance of various upper limb tasks is well established (Servos et al., 1992; Melmoth and Grant, 2006; Read et al., 2013; Granados-Delgado et al., 2024). Performance deficits have been reported in participants with reduced binocularity (Niechwiej-Szwedo et al., 2011; Grant and Conway, 2015; Kelly et al., 2021; Niechwiej-Szwedo, 2024; Rakshit et al., 2024; Verghese et al., 2016; Pardhan et al., 2011). This study is the first to examine the influence of a relatively mild visual impairment elicited by increased scatter of straylight on vision function (acuity, contrast sensitivity and stereoacuity), oculomotor function (phoria and fusional vergences), and motor performance (eye and hand kinematics) in healthy young participants. Results demonstrate that 0.4 Bangerter foils (in both eyes, FBE; and in only one eye, FDE) caused significant deterioration in visual acuity, contrast sensitivity, stereoacuity and break point of fusional vergences, but no impact on recovery of fusional vergences or phoria. In contrast to the hypothesis, the visual impairment due to Bangerter foils had a relatively small effect on prehension performance. Notably, this study reveals a significant relationship between phoria and peak reach velocity, with greater exophoria linked to slower task execution.

As hypothesized, and consistent with previous findings (Castro-Torres et al., 2021), our results demonstrated a reduction in visual acuity, contrast sensitivity, and stereoacuity that varied with the type of experimental manipulation. In line with previous results (Sheppard et al., 2021), 0.4 Bangerter foils applied to both eyes had the greatest effect on visual acuity and contrast sensitivity, specifically at higher spatial frequencies. It is important to keep in mind that the decrease in visual acuity and stereoacuity due to the foils was relatively mild in the current study. The results demonstrated a smaller reduction in visual acuity than that specified by the manufacturer of the Bangerter foil, which was claimed to degrade normal vision to approximately 0.4 logMAR. Nevertheless, our results are consistent with previous studies that also demonstrated similarly mild visual acuity (VA) impairment with the foils (Odell et al., 2008; Agervi et al., 2009). Consequently, the worst median stereoacuity value measured using the Frisby test in the impaired viewing condition was 40 arcsec, which is considered within a normal clinical range. Due to this limitation, the current study was not able to extend the work of Goodwin and colleagues which demonstrated a fundamental difference in the stereoacuity and VA relationship under conditions of monocularly and binocularly degraded acuity using spherical lenses (Goodwin and Romano, 1985). In that study, the mean stereoacuity appeared to be equally degraded by monocular and binocular deterioration in VA, within a range of 20/50 and 20/200 (Goodwin and Romano, 1985). Thus, future studies should use stronger Bangerter foils and increase the level of straylight deterioration to investigate the effects of reduced visual acuity on stereoacuity thresholds.

A greater reduction in stereoacuity was found when the interocular difference was introduced for the real depth (Frisby) test, whereas the Randot (polarized) test demonstrated smaller effects. This finding has been previously reported by other authors (Sheppard et al., 2021), using a polarized stereo test. Notably, Leske et al. concluded that results from different tests (i.e., real depth vs. polarized) are not interchangeable as these tests measure different aspects of stereopsis (Leske et al., 2006). The Frisby test might provide a more accurate measurement of real stereoacuity due to the absence of any dissociative mechanism.

The present study is the first to analyze the influence of increased straylight on phoria and fusional vergences. In the context of stereopsis, increased interocular acuity differences using Bangerter foils in one eye may destabilize the vergence system. Previous studies that used lens defocusing as an impairment methodology (Piano and O’Connor, 2013) found that increased interocular differences were associated with altered final phoria and the break and recovery points of the fusional vergences. The findings of the present study do not fully support this pattern of results. Our investigation revealed no impact of 0.4 Bangerter foils on phoria and fusional recovery points. Instead, the presence of foils over one eye was associated with lower break points of positive and negative fusional vergences. The increase of straylight may not modify the accommodation response as defocus blur does; however, the increase of interocular differences negatively affected the vergence responses.

Findings from the current study may have implications for understanding the efficacy of Bangerter treatment in amblyopia, which has been used to improve the visual functions of the amblyopic eye (Lee and Kim, 2021). Specifically, a more rapid visual acuity recovery with the Bangerter foils than with spectacles alone was found in patients with anisometropic amblyopia (Agervi et al., 2009). The underlying mechanisms to explain this effect remain to be established. It is well known that Bangerter foils degrade the image in one eye while still permitting parafoveal binocular interactions (Abrams et al., 2011). Our findings contribute to the existing literature by demonstrating no significant effect of Bangerter foils on phoria or fusional vergence recovery. Consequently, using 0.4 Bangerter foils seems to have little effect on the initial fusional state in case of healthy adults. Therefore, it is possible that the application of these foils serves to encourage fusional development, a notion that has been previously advanced by other researchers (Abrams et al., 2011). However, using a higher density foil could affect the fusion process.

As demonstrated by previous studies, vision provides an important input for planning and executing upper limb movements in a task-dependent manner (Niechwiej-Szwedo et al., 2023; Granados-Delgado et al., 2024; Sheppard et al., 2021). The present study used a bead threading task which allows detailed analyses of eye-hand coordination using a kinematic approach (Niechwiej-Szwedo et al., 2020; Niechwiej-Szwedo et al., 2021). This task was selected because it provides insight into the control of different movement components, such as reaching, grasping and placement (Niechwiej-Szwedo et al., 2020). Previous research demonstrated that grasping and threading are performed significantly more efficiently during binocular viewing, except in cases where binocularity is not fully compensated (i.e., due to high phoria and lower fusional vergences), where further investigation is needed. Moreover, mild and moderate disruption of stereoacuity (i.e., 200 arcsec and worse) using convex lenses was associated with prolonged grasp duration (Mroczkowski and Niechwiej-Szwedo, 2021). The current study extends these results by investigating the effect of induced visual impairment using Bangerter foils. The results demonstrated relatively minor effects on kinematic variables caused by the visual impairment due to straylight. Specifically, the total time required to complete the entire task was slightly prolonged when viewing with foils on both eyes compared to normal binocular viewing, which was mainly due to a longer threading duration. In contrast to previous studies with adults and children which demonstrated a significant contribution of stereoacuity to grasp planning and execution (Niechwiej-Szwedo et al., 2020; Servos et al., 1992; Melmoth and Grant, 2006; Gnanaseelan et al., 2014; Kelly et al., 2021; Niechwiej-Szwedo, 2024), grasp duration was not affected in the present study. This is most likely because the induced impairment was relatively weak and stereoacuity thresholds remained within the clinically accepted range for most participants. Our results add to the literature by demonstrating that a stereoacuity threshold of 50 arcsec is sufficient to support efficient grasp planning and execution.

Surprisingly, reach peak velocity was slightly but significantly higher when the foil was placed on the dominant eye (FDE) compared to binocular baseline (BB). Results from the linear mixed model confirmed it. We do not have an explanation for this effect. A study with a larger cohort is required to confirm these findings. On the other hand, linear mixed model analyses offered valuable insights into how individual differences in visual function influence visuomotor performance. Specifically, results revealed that lower exophoria and higher break point of fusional reserves were associated with higher peak velocity when reaching to the bead or towards the needle. Consistent with the effects on peak velocity, higher exophoria was associated with longer reach duration and prolonged total time in the execution of the bead threading task. These results indicate that the individual’s state of binocular vergence impacts the ability to plan reaching movements to a greater extent than the induced mild visual impairment using Bangerter foils. This underscores the crucial role of binocular vergence function in the performance of near motor tasks in adults with normal vision. Thus, the study has important implications for designing and interpreting experiments that assess visuomotor performance in the peripersonal space.

The current findings are not fully consistent with a previous study that found larger effects of induced visual deterioration using 0.3 logMAR Bangerter foils on the performance of water pouring and a pegboard task (Sheppard et al., 2021). In that study, the lowest visual acuity values (0.3 logMAR) were recorded for the visual condition with the foils in both eyes and poorest stereopsis (greater than 250 arcsec) with the foil in only one eye. The accuracy of water pouring, performance on the Purdue task, and on the Clinical Kinematic Assessment Tool were all found to be negatively affected when the test was performed using the foils placed on both eyes (Sheppard et al., 2021). These larger effects could be attributed to the increased task complexity and heterogeneity. In contrast, the induced vision deterioration with an increase in straylight in the current study had a relatively small effect on the performance of the bead threading task. Although the bead threading task requires high precision for grasping the small beads and placing them on the needle, the task was highly repetitive because the same bead was used, and the location was not varied across trials. This lack of variation led to stereotypical movements which did not require processing new sensory inputs to program a motor response on each trial. The null effect due to vision impairment on motor performance suggests that planning and executing the same movement across a number of trials does not require high acuity vision and could rely on preprogrammed motor commands and haptic feedback.

The present study has some limitations. First, the visual deterioration that Bangerter foils induced in each participant was variable, which contributed to the heterogeneity of the effects on motor performance. Future research could explore the impact of visual impairment by simulating the same level of acuity, contrast sensitivity or stereoacuity deficits in all participants. This could be achieved by using different levels of Bangerter foils to obtain the same visual impairment across participants. Given the small deficits caused by the foils in this study, future research should implement a larger deterioration leading to a higher level of stereopsis impairment. Second, the visual deterioration induced in the present study was relatively short, less than 1 h. Investigating the effects after a longer period of visual impairment could reveal long-term neural adaptation, which might be more relevant from a clinical perspective. Finally, the current study offers valuable insights into the role of the vergence system in upper limb reaching movements; however, a larger sample size is warranted to strengthen the findings derived from the mixed model analysis. Further research is required to translate these results into real-world applications, such as investigating the implications of phoria in everyday tasks.

## 5 Conclusion

In conclusion, inducing a short-term visual deterioration by reducing contrast sensitivity at high spatial frequencies without a corresponding deterioration in stereopsis had a relatively small effect on the performance of a bead threading task. Importantly, this study provides evidence that individual differences in binocular functions—such as phoria and fusional vergence—significantly influence reaching performance. It is important to note that these results cannot be extrapolated to patients with amblyopia, cataracts and/or eye conditions that involve a decrease in transparency and therefore an increase in straylight over a long period of time. However, the results may be comparable to those observed in patients who suddenly develop a condition of this nature, such as secondary cataract or corneal edema. In the context of amblyopia treatment using Bangerter foils, the findings from the present study may help elucidate the mechanisms underlying their effectiveness.

## Data Availability

Data will be available from the corresponding author on reasonable request.
