# Supplementary material for: Contribution of binocular visual function and its impairment by Bangerter foils to the performance of a precision reaching, grasping and placing task in healthy adults
Source: Front Neurosci. 2025 Dec 17;19:1708514. doi: 10.3389/fnins.2025.1708514 (PMC12753513; doi:10.3389/fnins.2025.1708514)
Supplement: Supplementary file 1 [file Table_1.docx]

**Supplementary material**

| **Eye-Hand Latency Difference** | |  |  |  |  |
| --- | --- | --- | --- | --- | --- |
| **Parameter** | **Coefficient** | **SE** | **t-statistic** | **p-value** | **[95% CI]** |
| **Viewing condition** |  |  |  |  |  |
| FDE | 23.468 | 22.295 | 1.053 | 0.297 | [(-21.230) ‒ 68.166] |
| FBE | 63.960 | 37.533 | 1.704 | 0.094 | [(-11.289) ‒ 139.209] |
| BB | ‒ | ‒ | ‒ | ‒ | ‒ |
| **Visual functions** |  |  |  |  |  |
| CS Mean | 0.482 | 0.348 | 1.386 | 0.171 | [(-0.215) ‒ 1.178] |
| VA Near | -101.019 | 121.513 | -0.832 | 0.409 | [(-344.735) ‒ 142.505] |
| Phoria Near | 0.453 | 2.803 | 0.162 | 0.872 | [(-5.166) ‒ 6.072] |
| Stereoacuity (Frisby) | -0.521 | 0.377 | -1.384 | 0.172 | [(-1.277) ‒ 0.234] |
| PFV Break point | -0.651 | 0.941 | -0.692 | 0.492 | [(-2.538) ‒ 1.236] |
| NFV Break point | -3.222 | 2.1153 | -1.523 | 0.134 | [(-7.463) ‒ 1.019] |
| Intercept | 147.723 | 64.316 | 2.297 | **0.026*** | [18.776 ‒ 276.670] |
| Bayesian information criterion | | | | | 1676.862 |

| **Reach-to-bead duration** | |  |  |  |  |  |
| --- | --- | --- | --- | --- | --- | --- |
| **Parameter** | **Coefficient** | **SE** | **df** | **t-statistic** | **p-value** | **[95% CI]** |
| **Viewing condition** | |  |  |  |  |  |
| FDE | -29.789 | 11.269 | 28.872 | -2.644 | **0.013*** | [(-52.840) ‒ (-6.738)] |
| FBE | -6.922 | 18.947 | 40.205 | -0.365 | 0.717 | [(-45.209) ‒ 31.366] |
| BB | ‒ | ‒ | ‒ | ‒ | ‒ | ‒ |
| **Visual functions** |  |  |  |  |  |  |
| CS Mean | -0.011 | 0.179 | 44.686 | -0.064 | 0.949 | [(-0.373) ‒ 0.350] |
| VA Near | -24.998 | 57.760 | 34.460 | -0.433 | 0.668 | [(-142.323) ‒ 92.327] |
| Phoria Near | -4.450 | 1.226 | 28.103 | -3.516 | **0.002*** | [(-7.042) ‒ (-1.858)] |
| Stereoacuity (Frisby) | 0.300 | 0.182 | 28.501 | 1.649 | 0.110 | [(-0.072) ‒ 0.673] |
| PFV Break point | -0.657 | 0.471 | 46.473 | -1.395 | 0.170 | [(-1.606) ‒ 0.291] |
| NFV Break point | -1.369 | 1.073 | 45.323 | -1.275 | 0.209 | [(-3.530) ‒ 0.792] |
| Intercept | 503.003 | 33.447 | 44.489 | 15.039 | **< 0.001**** | [435.616 ‒ 570.389] |
| Akaike information criterion | | 593.016 | Bayesian information criterion | | | 606.939 |
| **Reach-to-Bead Peak Velocity** | |  |  |  |  |  |
| **Parameter** | **Coefficient** | **SE** | **Df** | **t-statistic** | **p-value** | **[95% CI]** |
| **Viewing condition** | |  |  |  |  |  |
| FDE | 37.687 | 17.462 | 27.244 | 2.158 | **0.040*** | [1.873 ‒ 73.502] |
| FBE | -27.337 | 29.289 | 37.172 | -0.933 | 0.357 | [(-86.672) ‒ 31.998] |
| BB | ‒ | ‒ |  | ‒ | ‒ | ‒ |
| **Visual functions** |  |  |  |  |  |  |
| CS Mean | -0.244 | 0.281 | 42.675 | -0.868 | 0.390 | [(-0.810) ‒ 0.323] |
| VA Near | 89.335 | 90.132 | 32.041 | 0.991 | 0.329 | [(-94.249) ‒ 272.919] |
| Phoria Near | 7.460 | 2.065 | 30.698 | 3.612 | **0.001*** | [3.247 ‒ 11.674] |
| Stereoacuity (Frisby) | -0.030 | 0.290 | 29.969 | -0.104 | 0.918 | [(-0.623) ‒ 0.562] |
| PFV Break point | 1.892 | 0.766 | 46.648 | 2.470 | **0.017*** | [0.351 ‒ 3.432] |
| NFV Break point | 1.907 | 1.651 | 41.834 | 1.155 | 0.255 | [(-1.424) ‒ 5.238] |
| Intercept | 705.826 | 53.065 | 46.678 | 13.301 | **< 0.001**** | [599.054 ‒ 812.599] |
| Akaike information criterion | | 643.853 | Bayesian information criterion | | | 657.776 |

| **Grasp Duration** | |  |  |  |  |
| --- | --- | --- | --- | --- | --- |
| **Parameter** | **Coefficient** | **SE** | **t-statistic** | **p-value** | **[95% CI]** |
| **Viewing condition** | |  |  |  |  |
| FDE | 3.887 | 24.726 | 0.157 | 0.876 | [(-45.686) ‒ 53.459] |
| FBE | 63.197 | 39.384 | 1.605 | 0.114 | [(-15.762) ‒ 142.157] |
| BB | ‒ | ‒ | ‒ | ‒ | ‒ |
| **Visual functions** | |  |  |  |  |
| CS Mean | 0.368 | 0.3701 | 0.994 | 0.325 | [(-0.374) ‒ 1.110] |
| VA Near | -127.293 | 130.439 | -0.976 | 0.333 | [(-388.808) ‒ 134.222] |
| Phoria Near | -2.661 | 3.059 | -0.870 | 0.388 | [(-8.795) ‒ 3.472] |
| Stereoacuity (Frisby) | 0.026 | 0.416 | 0.062 | 0.951 | [(-0.807) ‒ 0.859] |
| PFV Break point | -1.218 | 0.987 | -1.235 | 0.222 | [(-3.197) ‒ 0.760] |
| NFV Break point | 0.807 | 2.348 | 0.344 | 0.732 | [(-3.899) ‒ 5.514] |
| Intercept | 138.555 | 67.237 | 2.061 | **0.044*** | [3.753 ‒ 273.357] |
| Bayesian information criterion | | | | | 1694.844 |

| **Reach-to-Needle Duration** | |  |  |  |  |  |
| --- | --- | --- | --- | --- | --- | --- |
| **Parameter** | **Coefficient** | **SE** | **Df** | **t-statistic** | **p-value** | **[95% CI]** |
| **Viewing condition** | |  |  |  |  |  |
| FDE | -27.679 | 10.007 | 27.016 | -2.766 | **0.010*** | [(-48.212) ‒ (-7.146)] |
| FBE | -51.271 | 17.655 | 43.911 | -2.904 | **0.006*** | [(-86.853) ‒ (-15.689)] |
| BB | ‒ | ‒ | ‒ | ‒ | ‒ | ‒ |
| **Visual functions** |  |  |  |  |  |  |
| CS Mean | -0.475 | 0.164 | 44.955 | -2.899 | **0.006*** | [(-0.805) ‒ (-0.145)] |
| VA Near | 49.958 | 53.828 | 37.209 | 0.928 | 0.359 | [(-59.088) ‒ 159.003] |
| Phoria Near | -5.185 | 0.991 | 25.136 | -5.231 | **< 0.001**** | [(-7.226) ‒ (-3.144)] |
| Stereoacuity (Frisby) | 0.170 | 0.148 | 20.764 | 1.149 | 0.263 | [(-0.138) ‒ 0.479] |
| PFV Break point | -0.252 | 0.399 | 37.426 | -0.630 | 0.532 | [(-1.060) ‒ 0.557] |
| NFV Break point | -2.125 | 0.977 | 44.375 | -2.175 | **0.035*** | [(-4.093) ‒ 0.156] |
| Intercept | 546.633 | 31.185 | 49.042 | 17.528 | **< 0.001**** | [483.965 ‒ 609.301] |
| Akaike information criterion | | 581.203 | Bayesian information criterion | | | 595.126 |
| **Reach-to-Needle Peak Velocity** | |  |  |  |  |  |
| **Parameter** | **Coefficient** | **SE** | **Df** | **t-statistic** | **p-value** | **[95% CI]** |
| **Viewing condition** | |  |  |  |  |  |
| FDE | 36.193 | 27.200 | 23.971 | 1.331 | 0.196 | [(-19.948) ‒ 92.335] |
| FBE | 25.179 | 45.458 | 20.835 | 0.554 | 0.586 | [(-69.402) ‒ 119.760] |
| BB | ‒ | ‒ | ‒ | ‒ | ‒ | ‒ |
| **Visual functions** |  |  |  |  |  |  |
| CS Mean | 0.547 | 0.436 | 26.231 | 1.253 | 0.221 | [(-0.350) ‒ 1.443] |
| VA Near | -10.729 | 139.091 | 17.639 | -0.077 | 0.939 | [(-303.379) ‒ 281.920] |
| Phoria Near | 13.062 | 2.930 | 22.421 | 4.458 | **< 0.001**** | [6.992 ‒ 19.131] |
| Stereoacuity (Frisby) | -0.209 | 0.433 | 20.487 | -0.483 | 0.634 | [(-1.111) ‒ 0.692] |
| PFV Break point | 1.070 | 1.152 | 28.309 | 0.928 | 0.361 | [(-1.290) ‒ 3.429] |
| NFV Break point | 2.930 | 2.542 | 27.868 | 1.153 | 0.259 | [(-2.278) ‒ 8.139] |
| Intercept | 716.387 | 82.945 | 30.441 | 8.637 | **< 0.001**** | [547.095 ‒ 885.680] |
| Akaike information criterion | | 648.451 | Bayesian information criterion | | | 698.374 |

| **Thread duration** | |  |  |  |  |
| --- | --- | --- | --- | --- | --- |
| **Parameter** | **Coefficient** | **SE** | **t-statistic** | **p-value** | **[95% CI]** |
| **Viewing condition** | |  |  |  |  |
| FDE | -0.887 | 31.772 | -0.028 | 0.978 | [(-64.587) ‒ 62.813] |
| FBE | 79.069 | 52.986 | 1.492 | 0.141 | [(-27.162) ‒ 185.300] |
| BB | ‒ | ‒ | ‒ | ‒ | ‒ |
| **Visual functions** |  |  |  |  |  |
| CS Mean | -0.030 | 0.503 | -0.060 | 0.952 | [(-1.039) ‒ 0.978] |
| VA Near | -132.202 | 167.310 | -0.790 | 0.433 | [(-467.639) ‒ 203.235] |
| Phoria Near | -1.430 | 3.465 | -0.413 | 0.681 | [(-8.377) ‒ 5.517] |
| Stereoacuity (Frisby) | 0.346 | 0.529 | 0.654 | 0.516 | [(-0.715) ‒ 1.407] |
| PFV Break point | -0.277 | 1.314 | -0.211 | 0.834 | [(-2.911) ‒ 2.357] |
| NFV Break point | -2.450 | 2.915 | -0.840 | 0.404 | [(-8.295) ‒ 3.395] |
| Intercept | 396.407 | 93.297 | 4.249 | **< 0.001**** | [209.358 ‒ 583.456] |
| Bayesian information criterion | | | | | 1722.126 |

| **Total Time** |  |  |  |  |  |  |
| --- | --- | --- | --- | --- | --- | --- |
| **Parameter** | **Coefficient** | **SE** | **Df** | **t-statistic** | **p-value** | **[95% CI]** |
| **Viewing condition** | |  |  |  |  |  |
| FDE | -49.984 | 49.586 | 25.436 | -1.008 | 0.323 | [(-152.021) ‒ 52.052] |
| FBE | 78.133 | 87.562 | 34.874 | 0.892 | 0.378 | [(-99.652) ‒ 255.917] |
| BB | ‒ | ‒ |  | ‒ | ‒ | ‒ |
| **Visual functions** | |  |  |  |  |  |
| CS Mean | -0.491 | 0.820 | 40.496 | -0.599 | 0.553 | [(-2.148) ‒ 1.166] |
| VA Near | -240.282 | 282.187 | 31.258 | -0.851 | 0.401 | [(-815.613) ‒ 335.050] |
| Phoria Near | -14.366 | 5.238 | 29.251 | -2.743 | **0.010*** | [(-25.075) ‒ (-3.657)] |
| Stereoacuity (Frisby) | 0.428 | 0.804 | 25.216 | 0.532 | 0.599 | [(-1.228) ‒ 2.084] |
| PFV Break point | -3.339 | 2.180 | 48.201 | -1.531 | 0.132 | [(-7.723) ‒ 1.044] |
| NFV Break point | -8.079 | 4.680 | 36.788 | -1.726 | 0.093 | [(-17.564) ‒ 1.405] |
| Intercept | 1710.443 | 155.607 | 46.775 | 10.992 | **< 0.001**** | [1397.362‒ 2023.523] |
| Akaike information criterion | | 757.363 | Bayesian information criterion | | | 771.286 |
